# Supplementary material for: Structural basis of organic cation transporter-3 inhibition
Source: Nat Commun. 2022 Nov 7;13:6714. doi: 10.1038/s41467-022-34284-8 (PMC9640557; doi:10.1038/s41467-022-34284-8)
Supplement: Supplementary file 3 — Reporting Summary [file 41467_2022_34284_MOESM3_ESM.pdf]

nature portfolio

Corresponding author(s): Thomas Stockner, Volodymyr M. Korkhov, Harald H. Sitte

Last updated by author(s): Sep 27, 2022

Reporting Summary

Nature Portfolio wishes to improve the reproducibility of the work that we publish. This form provides structure for consistency and transparency in reporting. For further information on Nature Portfolio policies, see our Editorial Policies and the Editorial Policy Checklist.

Statistics

For all statistical analyses, confirm that the following items are present in the figure legend, table legend, main text, or Methods section.

n/a	Confirmed	
☐	☒	The exact sample size (*n*) for each experimental group/condition, given as a discrete number and unit of measurement
☐	☒	A statement on whether measurements were taken from distinct samples or whether the same sample was measured repeatedly
☐	☒	The statistical test(s) used AND whether they are one- or two-sided<br>*Only common tests should be described solely by name; describe more complex techniques in the Methods section.*
☒	☐	A description of all covariates tested
☒	☐	A description of any assumptions or corrections, such as tests of normality and adjustment for multiple comparisons
☐	☒	A full description of the statistical parameters including central tendency (e.g. means) or other basic estimates (e.g. regression coefficient) AND variation (e.g. standard deviation) or associated estimates of uncertainty (e.g. confidence intervals)
☐	☒	For null hypothesis testing, the test statistic (e.g. *F*, *t*, *r*) with confidence intervals, effect sizes, degrees of freedom and *P* value noted<br>*Give P values as exact values whenever suitable.*
☒	☐	For Bayesian analysis, information on the choice of priors and Markov chain Monte Carlo settings
☐	☒	For hierarchical and complex designs, identification of the appropriate level for tests and full reporting of outcomes
☒	☐	Estimates of effect sizes (e.g. Cohen's *d*, Pearson's *r*), indicating how they were calculated

Our web collection on statistics for biologists contains articles on many of the points above.

Software and code

Policy information about availability of computer code

Data collection	Live Acquisition software 2.5.0.21<br>Relion 3.1.26
Data analysis	Fiji ImageJ 1.53c1<br>Offline Analysis 2.5.0.2<br>GraphPad Prism 9.2.0<br>RStudio (2021.09.2)<br>MotionCorr 1.1.0<br>Coot9<br>SwissModel<br>AlphaFold (AF-O75751-F1)<br>MolProbity<br>Phenix 1.16-3549<br>PyMOL 2.5.2<br>ChimeraX 1.2.5<br>Gromacs 2021

For manuscripts utilizing custom algorithms or software that are central to the research but not yet described in published literature, software must be made available to editors and reviewers. We strongly encourage code deposition in a community repository (e.g. GitHub). See the Nature Portfolio guidelines for submitting code & software for further information.

Data

Policy information about availability of data

All manuscripts must include a data availability statement. This statement should provide the following information, where applicable:

- Accession codes, unique identifiers, or web links for publicly available datasets
- A description of any restrictions on data availability
- For clinical datasets or third party data, please ensure that the statement adheres to our policy

The atomic coordinates and structure factors have been deposited in the Protein Data Bank (7ZH0, 7ZHA, 7ZH6); the density maps have been deposited in the Electron Microscopy Data Bank (EMD-14716, EMD-14728, EMD-14725). Data supporting the findings of this study are available within the article and its Supplementary Information Files and from the corresponding authors upon reasonable request.

Field-specific reporting

Please select the one below that is the best fit for your research. If you are not sure, read the appropriate sections before making your selection.

☒ Life sciences ☐ Behavioural & social sciences ☐ Ecological, evolutionary & environmental sciences

For a reference copy of the document with all sections, see nature.com/documents/nr-reporting-summary-flat.pdf

Life sciences study design

All studies must disclose on these points even when the disclosure is negative.

Sample size	No sample size calculations were necessary for this study. For in vitro assays, at least three biologically independent experiments, in triplicate, were conducted on separate days.
Data exclusions	Rarely extreme outliers in vitro assays were cautiously excluded, otherwise data was kept as complete as possible.
Replication	Experiments were at least conducted in three individual, biologically independent assays, in triplicate if appropriate. Rarely attempts at replication were unsuccessful and in such cases experiments were repeated two more times before excluding any gathered data.
Randomization	Genetic variants expressed in HEK293 cells that were tested in vitro were assigned a code and tested in a blinded manner in all assays. To accomplish blinding, the cell lines were randomly given a code.
Blinding	Analysis of genetic variants expressed in HEK293 cells that were tested in vitro or imaged were assigned a code was conducted in a blinded manner. For MD- or cryo-EM approaches, blinding was not possible and/or necessary.

Reporting for specific materials, systems and methods

We require information from authors about some types of materials, experimental systems and methods used in many studies. Here, indicate whether each material, system or method listed is relevant to your study. If you are not sure if a list item applies to your research, read the appropriate section before selecting a response.

Materials & experimental systems

n/a	Involved in the study	
☐	☒	Antibodies
☐	☒	Eukaryotic cell lines
☒	☐	Palaeontology and archaeology
☒	☐	Animals and other organisms
☐	☒	Human research participants
☒	☐	Clinical data
☒	☐	Dual use research of concern

Methods

n/a	Involved in the study	
☒	☐	ChIP-seq
☒	☐	Flow cytometry
☒	☐	MRI-based neuroimaging

Antibodies

Antibodies used	Rabbit anti-GFP polyclonal antibody (A6455, Thermo Fisher)
Validation	Validated by manufacturer.

Eukaryotic cell lines

Policy information about cell lines

Cell line source(s)	ATCC, human embryonic kidney cells 293, human embryonic kidney cells 293F
Authentication	No authentication was performed.
Mycoplasma contamination	Mycoplasma contamination was regularly tested and could be ruled out for all cell lines used in this study.
Commonly misidentified lines (See ICLAC register)	None

Human research participants

Policy information about studies involving human research participants

Population characteristics	The exome sequencing data used in this study is a nested case-control sample, which is part of the integrated psychiatric research (iPSYCH) consortium's first phase genotyping of a nation-wide Danish birth cohort that has been described in detail previously (Pedersen, C.B. et al. Mol Psychiatry 23, 6-14 (2018)). The iPSYCH2012 case-cohort includes individuals diagnosed with schizophrenia, mood disorders, bipolar affective disorder, autism and attention-deficit/hyperactivity disorder which were identified through linkage between Danish population-based registers. Controls are a random sample of the same population that supplied the cases. A subset of 19,851 samples were exome sequenced using the Illumina Nextera Rapid Capture kit at 20x average depth. Procedures for exome sequencing, sample-and variant have been detailed in (Herborg, F. et al. JCI Insight 6(2021).). Fishers-exact test was used to compare carrier frequencies of coding SLC22A3 variants in cases and controls.
Recruitment	The study base of the iPSYCH2012 cohort include all singleton births with known mothers born between 1 of May 1981 and 31 of December 2005, who were alive and resided in Denmark at their first birthday. The diagnosis are drawn from Danish national registers and dried blood samples are obtained from the Danish Neonatal Biobank. Accordingly, self-selection bias is not possible and the only selection bias involved is that the data solely consists of Danish patients of a relatively young age.
Ethics oversight	The Danish Scientific Ethics Committee, the Danish Data Protection Agency and the Danish Neonatal Screening Biobank Steering Committee approved the iPSYCH study. Informed consent is not required for register-based research in Denmark.

Note that full information on the approval of the study protocol must also be provided in the manuscript.
